# Prognostic Biopsy of Choroidal Melanoma Before and After Ruthenium-106 Plaque Brachytherapy: Impact on Success of Cytogenetic Analysis

**DOI:** 10.3390/cancers17122057

**Published:** 2025-06-19

**Authors:** Keri McLean, Helen Kalirai, Muhammad H. Amer, Bertil Damato, Sarah E. Coupland, Heinrich Heimann, Rumana N. Hussain

**Affiliations:** 1 Department of Eye and Vision Science, Institute of Life Course and Medical Science, University of Liverpool, Liverpool L7 8TX, UK; hkalirai@liverpool.ac.uk (H.K.); amelie@liverpool.ac.uk (S.E.C.); heinrich.heimann@gmail.com (H.H.); 2 Liverpool Ocular Oncology Centre, Liverpool University Hospitals Trust, Liverpool L7 8XP, UK; 3 Liverpool Clinical Laboratories, Liverpool University Hospitals Trust, Liverpool L7 8XP, UK; 4 School of Medicine, Institute of Life Course and Medical Science, University of Liverpool, Liverpool L69 3GE, UK; hlmamer@liverpool.ac.uk; 5 Ocular Oncology Service, Moorfields Eye Hospital, London EC1V 2PD, UK; bertil.damato@gmail.com

**Keywords:** ruthenium brachytherapy, choroidal melanoma, biopsy, genetics, cytology

## Abstract

**Background/Objectives:** To determine if the results of cytogenetic analyses of choroidal melanoma biopsies after ruthenium-106 plaque brachytherapy (RPB) are affected by this procedure. **Methods**: A retrospective study was conducted on 368 patients with choroidal melanoma treated with RPB who underwent cytogenetic testing at the Liverpool Ocular Oncology Centre (LOOC) between May 2012 and November 2024. Data on demographics, tumor characteristics, treatment date, biopsy timing (pre- or post-RPB), and cytogenetic results were extracted from the LOOC database. Statistical analysis included descriptive statistics, binary, and multinomial logistic regression to assess associations between biopsy timing and biopsy success rates. **Results**: Biopsies were performed before RPB in 58.7% (216/368) cases, and post-PBR in 41.3%. Cytomorphological identification and molecular genetic testing were successful in 96.4% and 85.1% cases, respectively. Timing of biopsy, patient demographics, and tumor characteristics did not significantly influence cytogenetic test outcomes. Molecular testing could not be performed on 6.8% (25/368) cases as the DNA was insufficient in these samples. Genetic testing success slightly declined beyond three months post-RPB, though a few cases had delayed biopsy (n = 8). Pre-RPB biopsies more frequently demonstrated monosomy 3, whereas post-RPB biopsies had higher rates of disomy 3 (χ^2^, *p* < 0.05). **Conclusions**: Prognostic biopsies post-RPB provide reliable cytomorphological and molecular genetic results using MLPA or MSA. Test failure is not significantly influenced by biopsy timing, patient or tumor characteristics, biopsy modality, or genetic technique. Insufficient DNA yield remains a key limitation, emphasizing the importance of obtaining adequate tissue samples. Biopsies within three months are preferable to optimize success in molecular testing.

## Simple Summary

Choroidal melanoma is a rare eye cancer, and biopsies are often performed to predict how the disease may behave and to guide follow-up care. Some patients receive radiation treatment before the biopsy, raising concerns about whether this might affect the accuracy of genetic test results. This study looked at the largest group of patients to date to explore whether timing of the biopsy, especially in relation to radiation, affects test success. The results showed that biopsies remain reliable whether they are taken before or after radiation. Factors like patient age, tumor size, biopsy method, or testing technique did not significantly influence test outcomes. However, biopsies performed within three months of radiation were slightly more successful, particularly in smaller tumors. The most important factor for success was obtaining enough high-quality DNA, highlighting the need for surgeons to collect adequate tissue.

## 1. Introduction

Choroidal melanomas (CM) are the most common primary intraocular malignancy in adults, with a global incidence ranging from 2 to 8 per million person-years [1]. Almost 50% of patients develop metastatic disease, which almost always involves the liver, and which is usually fatal [2,3,4]. Metastatic disease occurs almost exclusively in patients whose tumor shows chromosome 3 loss, with or without changes in chromosome 8q, and/or class 2 gene expression profile [5,6,7,8,9]. There is currently no curative treatment for metastatic disease; however, tumor biopsy for cytogenetic analysis is increasingly being offered to these patients for several reasons. First, survival prognostication is enhanced by multivariable analysis of anatomical, histologic, and genetic predictors [10]. Second, most patients understandably want to know their prognosis [11]. Finally, accurate prognostication enables patients with a good survival probability to be reassured while targeting systemic surveillance and, in future, systemic adjuvant therapy at those likely to develop metastatic disease.

Most CM patients in the UK are treated with either proton beam radiotherapy (PBR) or ruthenium-106 plaque brachytherapy (RPB). Prognostic biopsies are performed by a transscleral or transretinal approach [12]. Pre-radiotherapy biopsy can cause complications, such as vitreous hemorrhage, which may result in postponement of radiotherapy, subconjunctival seeding, and, in patients with metastatic disease, concerns that the biopsy has caused this problem. For these reasons, at the Liverpool Ocular Oncology Centre, prognostic tumor biopsy is performed during plaque or tantalum marker insertion or after completion of the radiotherapy. We occasionally perform diagnostic biopsies prior to RPB when clinically indicated. The biopsy samples undergo histological and genetic analysis (i.e., multiplex ligation dependent probe amplification (MLPA) and/or microsatellite analysis (MSA)) [13], with the latter being dependent on DNA concentration and quality.

In our practice, RPB is typically offered to patients with small-to-medium sized CMs less than 6 mm in thickness. The plaque is sutured to the sclera overlying the tumor and removed one to six days later, after delivering minimum doses of 350 Gy and 90 Gy to the sclera and tumor apex, respectively. Although prognostic biopsy is typically performed either at the time of plaque insertion or removal, there have been occasions where prognostic biopsies have taken place prior to RPB or months or years later.

The aim of this study was to determine if the results of cytogenetic analyses of choroidal melanoma biopsies after RPB are affected by this procedure.

## 2. Materials and Methods

An in-house electronic database search was conducted to identify all patients treated with RPB for choroidal melanoma at the Liverpool Ocular Oncology Centre (LOOC) between 1 May 2012, and 4 November 2024, and whose tumor was analyzed genetically. Patients were excluded if the cytogenetic studies were performed on tumor samples harvested from subsequent enucleation specimens.

The following patient demographics and tumor characteristics were extracted from the LOOC database: patient’s age and sex; tumor location, ultrasound diameter and thickness; and secondary effects, including subretinal fluid (SRF), drusen, and lipofuscin. Details of treatment and pathology analysis included dates of plaque insertion and biopsy procedure, as well as biopsy method, specimen type, cell type, genetic technique (MLPA, MSA or both) and status of chromosomes 1, 3, 6 and 8. Methods of transscleral and transretinal biopsies as well as MLPA and MSA, post-July 2012, have previously been published [12,13,14]. The biopsy method was selected according to tumor size, location, treatment method, and surgeon’s preference. Pre- and post-equatorial biopsies tended to be performed transsclerally or transretinally, respectively. Transretinal biopsies were performed under local anesthesia with a 25-gauge vitrector using a 3-port sutureless vitrectomy kit, as previously described [14]. In the early years of the study, transscleral biopsies were performed using fine-needle aspiration biopsy (FNAB) with a small gauge needle (25–30G) [15]. Over time, our practice transitioned to using Essen forceps under a lamellar scleral flap, as this approach improved DNA yield by allowing direct tumor visualization [13]. Diathermy and 70% ethanol applied with a cotton bud were used to prevent seeding, followed by tissue glue to ensure watertight wound closure [13,15]. The National Health Service (NHS) Cancer Registry, which feeds into the LOOC database, was used to extract additional information on date of detection of metastatic disease, and date and cause of death.

Biopsy was categorized as successfully yielding a cytogenetic result if it could be classified as epithelioid, mixed, spindle or melanoma of unspecified cell type. Molecular genetics was classified as successful if sufficient DNA was obtained to undertake either MLPA or MSA and genetic information, i.e., loss, normal or gain could be obtained for the chromosomes 1p, 3, 6 and 8 when tested.

Biopsy was categorized according to whether it was performed on a date before RPB, at the time of plaque insertion, within one month of completion of RPB, two to three months after RPB, three to six months after RPB, and more than six months after RPB.

Statistical analysis was performed in SPSS Version 28 (IBM Corp., Armonk, NY, USA). This included descriptive analysis, binary logistic regression, and multinomial logistic regression modeling.

## 3. Results

A total of 368 patients (49.2% male, 50.8% female, (χ^2^, *p* = 0.635)) were included in this study. Table 1 outlines the baseline characteristics of these patients, according to whether the biopsy was pre- or post-RPB. The mean age at the time of tumor diagnosis was 60.6 ± 13.2 years (range 21–92) with no difference between groups (Mann–Whitney, *p* = 0.393). Tumor laterality showed no difference between groups (50.8% right v 49.2% left; χ^2^ *p* = 0.635). The mean largest basal diameter (LBD) was 11.0 ± 2.7 mm (range, 1.1–18.4) and the mean tumor thickness was 3.3 mm (range, 0.6–7.2), as measured by ultrasound. Tumor thickness did not follow a normal distribution, with the pre-RPB patients having thicker tumors (Mann–Whitney U, *p* < 0.001). LBD followed a normal distribution, with the pre-RPB patients shown to have larger tumors (Independent *t*-test, *p* < 0.001). More patients in the pre-RPB group had pre-equatorial tumors compared to the post-RPB group (χ^2^, *p* < 0.001). SRF, drusen, and lipofuscin were documented as present in 48.8% (158/324), 13.1% (38/290) and 37.3% (118/316) tumors, respectively. SRF and lipofuscin at diagnosis were recorded in more cases in the post-RPB group (Fisher’s Exact, *p* < 0.001).

TMN stage T2, was the most common in all RPB patients analyzed (153/368; 41.6%). Post hoc testing revealed tumors with pre-RPB biopsy were more likely to have T3 TNM stage than post-RPB, and post-RPB tumor tended to have T1 staged tumors (Z-test adjusted Bonferroni, *p* < 0.05). The number of T2 and T4 tumors did not differ significantly between groups.

The number of days between plaque insertion and biopsy ranged from −49 days to +3652 days, with a median of 0 days (i.e., biopsy on the same day as plaque insertion). Significantly more patients had a biopsy taken before RPB (216/368; 58.7%) compared to after RPB (152/368; 41.3%) (Fisher’s Exact compared to expected even distribution, *p* = 0.022). Of those in the pre-RPB group, 13.0% (28/216) underwent biopsy prior to plaque insertion (different day) and 87.0% (188/216) had the biopsy performed during the plaque insertion. Of note, 12.2% (45/368) of biopsies were performed between days one to seven following plaque insertion, at the time of plaque removal. In the post-RPB group, 47.4% (72/152) had the biopsy within one month of RPB, 47.4% (72/152) had the biopsy two to three months after RPB, 3.3% (5/152) had the biopsy three to six months after RPB and 2.0% (3/152) had the specimen collected more than six months after RPB. The 368 biopsies were transretinal in 164 (44.6%), transscleral in 201 (54.6%), conjunctival in two cases (0.5%) and episcleral in one (0.3%) case. Transscleral biopsies were performed more in the pre-RPB group and transretinal more in the post-RPB group (Z-test adjusted Bonferroni, *p* < 0.05).

A successful cytomorphological identification was recorded in 96.4% (351/364) of the biopsies, thus the failure rate was 3.6% (13/364) (Table 2). There was no difference between success rates of pre- and post-RPB (Table 2; Fisher’s Exact, *p* = 0.252). When stratified by time from RPB to biopsy, the failure rates were consistently below 5% (Table 2), although the sample size after three months was very small (*n* = 8). The tumor cytomorphology was epithelioid or mixed cell type in 27.7% (101/364) of cases, spindle cell in 67.9% (247/364) and melanoma of unspecified cell type in 0.8% (3/354) of cases. There was no difference in cytomorphology results when comparing pre-RPB to post-RPB (χ^2^, *p* = 0.520). A binary logistic regression model was fitted to assess if there was a temporal relationship between RPB and the failure of cytomorphology analysis, as well as the impact of patient age, sex, longest basal diameter, tumor thickness and biopsy modality (i.e., transscleral, transretinal, etc.). There was no evidence that any of these factors were associated with failure of cytomorphological analysis (Nagelkerke R^2^ = 0.117, Hosmer and Lemeshow test, *p* = 0.932).

Molecular genetics was not performed on 25 (6.8%) biopsy samples as the DNA yield was too low to perform the tests, with no difference in this respect between pre- and post-plaque groups (χ^2^, *p* = 0.177). Of these, 17 (68.0%) were obtained via transscleral biopsy, the majority (64.7%; 11/17) performed between 2012 and 2014. MSA was performed more frequently than MLPA (MSA 79.1% vs. MLPA 14.1%) but there was no difference between pre- and post-RPB groups (χ^2^, *p* = 0.177). The overall failure rate of genetic analysis was defined as the inability to obtain a genetic result (i.e., chromosomal loss, disomy, or chromosomal gain) for any of the chromosomes tested (1p, 3, 6p/q or 8p/q), despite the presence of sufficient DNA to conduct the test. The overall failure rate was 14.9%, corresponding to a success rate of 85.1% (328/343) (Table 2). There was no difference between success rates of pre- and post-RPB (Table 2; χ^2^, *p* = 0.372). When stratified by time from RPB to biopsy, the success rates of molecular tests were over 85%, except for those samples taken between 2 and 3 months when the success rate dropped to 82.9% (58/70) (Table 2). Of note, the sample size after three months is small (*n* = 8).

A multinomial logistic regression model was fitted to assess if there was a temporal relationship between RPB and the failure of molecular genetic tests, as well as the impact of patient age, sex, longest basal diameter, tumor thickness, biopsy modality (i.e., transscleral, transretinal, etc.) and genetic technique performed (MLPA or MSA). None of these factors influenced the success of molecular genetic tests, although there was an acceptable fit of the data (χ^2^(710) = 704.8, *p* = 0.549).

Chromosome 3 status was determined in 343 patients (Table 3). The majority exhibited disomy 3 (61.2%, 210/343), while 21.6% (74/343) had monosomy 3 and 17.2% (59/343) experienced a test failure despite adequate DNA. Monosomy 3 was more frequently detected in the pre-RPB group, whereas disomy 3 was more prevalent in the post-RPB group (Z-test adjusted Bonferroni, *p* < 0.05). Chromosome 8p status was analyzed in 51 patients, revealing chromosome 8p loss in 13.7% (7/51), disomy in 58.8% (30/51), 8p gain in 15.7% (8/51), and test failure in 13.7% (7/51) despite adequate DNA for testing (Table 3). There was no difference between pre- and post-RPB groups (χ^2^, *p* = 0.622). Regarding chromosome 8q, a normal copy number was recorded in 51.0% (26/51) and 8q gain in 41.2% (21/51) with failure in 7.8% (4/51) despite sufficient DNA for analysis (Table 3). There was no difference between pre- and post-RPB groups (χ^2^, *p* = 0.440).

Median follow-up was 2.55 years (range 0–10.9 years), with 321 patients followed for more than one year, 81 patients for more than five years and one patient for over ten years. Metastatic disease was documented in 32 patients (8.7%, 32/368), with 23 cases (10.6%; 23/216) occurring in the pre-RPB group and nine cases (5.92%; 9/152) in the post-RPB group (Fisher’s Exact, *p* = 0.134). Biopsy technique also did not influence the occurrence of metastatic disease with 19 cases (9.45%; 19/201) recorded in patients who underwent transscleral biopsy and 13 cases (7.93%; 13/164) in those who underwent trans-retinal biopsy (Fisher’s Exact, *p* = 0.711). At the study closure, 40 patients (10.9%, 40/368) were recorded as deceased, with a higher proportion in the pre-RPB (16.8%; 31/185) compared to the post-RPB group (6.29%; 9/143) (Fisher’s Exact, *p* = 0.011). However, there was no difference when stratified by biopsy technique with 22 deaths (10.9%; 22/201) occurring in patients who underwent transscleral biopsy and 18 deaths (11.0%; 18/164) occurring in patients who underwent transretinal biopsy (Fisher’s Exact, *p* = 1.00).

Of the deceased patients, 10 (25.0%; 10/40) were confirmed to have died from metastatic melanoma, while the cause of death was unknown for 18 patients. One patient underwent a biopsy four days before RPB and was found to have monosomy 3. Seven patients had biopsies performed on the day of plaque insertion; of these, five were transscleral with monosomy 3, while two were transretinal, one exhibiting disomy 3 and the other had a genetic test failure despite having sufficient DNA. One patient underwent a biopsy at plaque removal, but the test failed due to insufficient DNA. Another patient underwent transretinal biopsy 42 days post-RPB and was found to have disomy 3.

Both patients with disomy 3 CM who died from metastatic melanoma were classified with T3 tumors at diagnosis. One exhibited spindle cell morphology, while the other was of unknown cell type. The latter demonstrated additional genetic alterations, including a gain of chromosome 6p but a normal 8q copy number.

## 4. Discussion

Prognostic biopsies have been offered to patients with choroidal melanoma as part of their holistic management at the Liverpool Ocular Oncology Centre for over a decade. To avoid multiple surgical procedures, most patients undergo a biopsy at the time of plaque insertion or removal. Less commonly, patients will have undergone biopsies prior to the radiotherapy, if the biopsy is performed for diagnostic purposes. In other patients the biopsy is performed months or years after the radiotherapy, particularly if tumor relapse is suspected.

It is important to understand if the timing of radiotherapy in relation to the biopsy impacts cytogenetic tests, particularly when these tests have failed. To our knowledge, this is the largest study assessing the temporal association between the failure of cytogenetic tests and the timing of biopsy in relation to ruthenium plaque brachytherapy.

In our study, more patients underwent biopsy before or on the same day as plaque insertion compared to after plaque removal (58.7% vs. 41.3%, *p* = 0.022). Notably, 51.1% (188/368) of patients had the biopsy performed during the same procedure as plaque insertion and 12.2% (45/368) were performed at plaque removal. Of these patients, 98.9% (186/188) had a transscleral biopsy. This preference for performing transscleral biopsies at the time of plaque insertion may reflect practical surgical considerations. At this stage, the area of interest is already exposed and accessible, and the surrounding conjunctiva and scleral tissues are generally firmer and easier to manipulate. In contrast, by the time of plaque removal, these tissues may have become more friable [16].

The pre- and post-RPB groups were comparable in age, sex, tumor laterality and presence of pre-operative drusen. There was a difference in mean tumor size between pre- and post-RPB groups. The post-RPB group had thinner tumors with a smaller LBD (Mann–Whitney U, *p* < 0.001 and independent *t*-test, *p* < 0.001, respectively). This is consistent with our clinical practice of biopsying smaller, more posterior tumors via a transretinal route during a second procedure.

The success rate of cytomorphological identification of choroidal melanoma specimens was high, 96.4 (351/364) and comparable with similar studies [13,17,18]. The success rate was slightly higher in the post-RPB group compared to pre-RPB (98.0% v 95.3%) although this was not significant (Fisher’s exact *p* = 0.252). Similarly to other literature, spindle cells were the predominant cell type identified among the whole study population (67.9%) followed by epithelioid or mixed (27.7%) [13]. There was no difference in the cytomorphology type identified between pre- and post-RPB group (χ^2^, *p* = 0.520). These findings suggest that cytomorphological identification following RPB is still reliable.

We demonstrated, using binary logistic regression modeling of 364 cytomorphological tests, that there was no temporal relationship between timing of biopsy with respect to RPB and the failure of cytomorphological tests. Furthermore, this modeling demonstrated that patient age, sex, longest basal diameter, tumor thickness and biopsy modality (i.e., transscleral, transretinal, etc.) did not significantly contribute to test failure. Our findings suggest that the timing of biopsies taken for cytomorphological analysis would ideally be within six months of RPB, and perhaps even earlier in smaller tumors where post-RPB tumor regression may make the tumor sampling more difficult.

Molecular genetic testing achieved a success rate of 79.3% (292/368). Of the 76 failed tests, 25 (32.9%) were attributed to insufficient DNA yield from the biopsy sample. Notably, 56.0% (14/25) of these inadequate samples originated from transscleral biopsies, the majority of which were performed between 2012 and 2014, before the adoption of Essen forceps in our practice. This technique has been shown to enhance DNA yield and improve the success of molecular genetic testing by enabling direct tumor visualization and facilitating the acquisition of larger tissue samples [13]. To optimize DNA yield, surgeons should be encouraged to obtain the maximum feasible tissue sample during biopsy, regardless of whether a transscleral or transretinal approach is used.

When sufficient DNA was present, the success rate of molecular genetic testing was relatively high, 85.1%, and comparable with similar studies [19,20]. There was no difference in success rates between pre- and post-RPB groups (χ^2^, *p* = 0.372). Multinomial logistic regression modeling demonstrated that there was no temporal relationship between RPB and the failure of cytomorphological tests. Likewise, age, sex, longest basal diameter, tumor thickness, biopsy modality (i.e., transscleral, transretinal, etc.) and genetic technique (MLPA, MSA or both) did not significantly contribute to test failure. Notably, the genetic testing success rate declined slightly after two months (82.9%, 58/70), highlighting the potential benefit of performing prognostic biopsies earlier.

From a prognostic perspective, failure to characterize chromosomes 3 and 8q may be particularly limiting, as these markers are integral to the Liverpool Uveal Melanoma Prognosticator Online (LUMPO3) algorithm which provides personalized 10-year survival probabilities for patients with CM and supports clinical decision-making regarding risk stratification and surveillance planning [21].

Regarding genetic results, there was no difference in the status of chromosomes 1, 6 and 8 between groups; however, chromosome 3 status was found to be significantly different between pre- and post-RPB groups (χ^2^, *p* < 0.001). More patients within the post-RPB group demonstrated disomy 3 (post-RPB: 70.3% v pre-RPB: 54.5%) compared to the pre-RPB group. This may be because significantly more tumors in the post-RPB were smaller and classed as stage T1, whereas significantly more pre-RPB tumors were larger and more advanced (T3). Monosomy 3 has been shown to be more frequent in T3 uveal melanomas [22].

Nevertheless, the higher frequency of normal chromosomes 1p, 3 and 8p/q in the post-radiotherapy group would suggest that ruthenium exposure does not cause chromosomal loss. This is corroborated by the literature, which suggests that pre-irradiated CM biopsies, post-irradiated biopsies can also be successfully tested by MSA and MLPA to provide genetic information suitable for prognostication [23]. Coupland et al. demonstrated concordance in the chromosome 3 status of four CM, which underwent MSA/MLPA both pre- and post-radiotherapy [23]. Similarly, Wackernagal et al. reported the same genetic results in five CM samples, which underwent array comparative genomic hybridization (CGH) both pre- and post-radiotherapy [24]. MSA has been shown to successfully determine chromosome 3 status in 348 patients, irrespective of their radiation status [20]. We have also previously shown that MLPA or MSA can be used to successfully distinguish between monosomy 3 and disomy 3 in biopsies taken following proton beam radiotherapy [25].

Genetic analysis of CM by other techniques, such as karyotyping and fluorescence in situ hybridization (FISH), have been reported as less successful or reliable post-radiotherapy. Dogrusöz et al. reported that karyotyping and/or FISH of 36 previously irradiated CMs (28 RPB, 5 PBR and 3 stereotactic) exhibited more frequent and complex chromosomal aberrations compared to non-radiated tumors [26]. Additionally, the failure rate was significantly higher in previously irradiated tumors [26]. In comparison to our study, those genetic studies were performed many months after radiotherapy (mean: 45.7 months) when tumor necrosis, shrinkage and inflammation was more evident, and when clonal expansion of any surviving melanoma cells may have occurred. Their results, therefore, cannot be extrapolated to our post-RPB group in which 94.8% (144/152) of patients had their biopsy within three months of RPB. Gold et al. reported successful gene expression profiling (GEP) in three patients’ post-radiotherapy. Two at 27 and 36 months after Iodine-125 plaque brachytherapy, and one at 19 months post-PBR [27]. To our knowledge the feasibility and success of GEP specifically following RPB has not been previously documented.

Complications of a prognostic biopsy were not specifically analyzed in this study; however, tumor seeding and subsequent metastasis remain recognized concerns, particularly when biopsies are performed prior to irradiation [15]. Importantly, we found no significant association between biopsy timing or technique and the development of metastatic disease. This supports the oncological safety of both transscleral and transretinal approaches for prognostic biopsy, consistent with existing literature [28,29,30]. This may reflect precautionary measures taken in our clinical practice to minimize seeding risk, including the use of diathermy, alcohol-soaked swabs, and tissue adhesive to seal the biopsy tract during transscleral procedures [15]. Additionally, placement of the ruthenium plaque over the tumor and biopsy site delivers radiation which would sterilize any seeded cells [15]. While these findings are reassuring, the small number of metastatic deaths, incomplete cause-of-death data and median follow-up of 2.55 years limited our ability to further assess melanoma-specific mortality.

Our findings indicate that biopsy timing does not affect cytogenetic test failure rates, metastatic risk, or all-cause mortality. This supports clinical flexibility in scheduling biopsies either before or after brachytherapy. Such flexibility may improve surgical workflow efficiency, especially in centers with constrained scheduling resources. In some cases, deferring biopsy until plaque removal may be more practical without compromising diagnostic yield or patient safety. Furthermore, post-radiotherapy biopsies may enable molecular or histopathological assessment of tumor response, which could inform future prognostic or therapeutic strategies.

## 5. Conclusions

In conclusion, we have demonstrated that prognostic biopsies for choroidal melanoma provide reliable cytomorphological and molecular genetic results using MLPA or MSA, regardless of prior irradiation. We have shown, within limits, that test failures are not impacted by time from RPB to biopsy, age, sex, tumor thickness, biopsy modality and genetic technique. Insufficient DNA yield remains a key limitation in molecular genetic testing, emphasizing the need for surgeons to obtain the maximum feasible tissue sample during biopsy. Our findings also suggest that biopsies for cytomorphological and genetic analysis should ideally be performed within three months of RPB, with earlier sampling preferred for smaller tumors to mitigate challenges related to post-RPB tumor regression and fibrosis.

## Figures and Tables

**Table 1:** Characteristics of the study population.

	Overall *n* = 368 (% Total Unless Stated)	Pre-RPB *n* = 216 (% Group Unless Stated)	Post-RPB *n* = 152 (% Group Unless Stated)	*p*-Value
**Sex**				0.635 *
Male	181 (49.2)	104 (48.1)	77 (50.7)	
Female	187 (50.8)	112 (51.9)	75 (49.3)	
**Age Mean (range)**	60.6 (21–92) years	61.0 (21–91)	60.0 (27–92)	0.393 ^ⴕ^
**Eye**				0.635 *
Right	187 (50.8)	112 (51.9)	75 (49.3)	
Left	181 (49.2)	104 (48.1)	77 (50.7)	
**Tumor thickness Mean** **±** **SD (Range) in mm**	3.3 ± 1.44 mm (0.6–7.2)	3.6 ± 1.4 mm (1.0–7.2)	3.0 ± 1.3 mm (0.6–6.6)	<0.001 ^ⴕ^
**LBD** **Mean ± SD (Range) in mm**	11.0 ± 2.7 mm (1.1–18.4)	11.4 ± 2.7 mm (1.1–17.6)	10.3 ± 2.5 mm (4.4–18.4)	<0.001 ^§^
**Tumor location (anterior margin)**				<0.001 *
Pre-equatorial	164/289 (56.7)	127/180 (70.6)	37/109 (33.9)	
Post-equatorial	125/289 (43.3)	53/180 (29.4)	72/109 (66.1)	
**Subretinal fluid**	158/324 (48.8)	69/189 (36.5)	89/135 (65.9)	<0.001 ^ⱡ^
**Drusen**	38/290 (13.1)	24/181 (13.3)	14/109 (12.8)	0.535 ^ⱡ^
**Lipofuscin**	118/316 (37.3)	45/185 (24.3)	73/131 (55.7)	<0.001 ^ⱡ^
**TNM Staging**				<0.001 *
T1	89 (24.2)	36 (16.7)	53 (34.9)	
T2	153 (41.6)	91 (42.1)	62 (40.8)	
T3	124 (33.7)	89 (41.2)	35 (23.0)	
T4	2 (0.5)	-	2 (1.3)	
**Number of days between biopsy and plaque**	Mean: 29.8 days (Range: −49 to 3652) Median: 0 days	Mean: −1.86 days (Range: −49 to 0) Median: 0 days	Mean: 74.9 days (Range: 1–3652) Median: 35 days	<0.001 *
Prior to plaque	28 (7.6)	28 (13.0)	-	
Same day plaque	188 (51.1)	188 (87.0)	-	
Within 1 month	72 (19.6)	-	72 (47.4)	
2–3 months	72 (19.6)	-	72 (47.4)	
3–6 months	5 (1.4)	-	5 (3.3)	
**More than 6 months**	3 (0.8)	-	3 (2.0)	
**Biopsy Technique**				<0.001 *
Transretinal	164 (44.6)	40 (18.5)	124 (81.6)	
Transscleral	201 (54.6)	174 (80.6)	27 (17.8)	
Conjunctival	2 (0.5)	2 (0.9)	-	
Episcleral	1 (0.3)	-	1 (0.7)	

RPB, Ruthenium plaque brachytherapy; LBD, largest basal diameter. Statistical comparisons were made between Pre-RPB and Post-RPB: * Chi-squared test; ^§^ Independent *t*-test; ^ⴕ^ Mann–Whitney U test; ^ⱡ^ Fisher’s Exact test.

**Table 2:** Failure rates of cytomorphology and molecular genetic testing stratified with respect to time from ruthenium plaque brachytherapy to biopsy.

	Cytomorphology % (*n*)		Genetics % (*n*)	
	All cases (*n* = 368)		All cases (*n* = 368)	
	Not performed in 1.1% (4/368).		Failed due to insufficient DNA yield from biopsy in 6.8% (25/368)	
	**Pre-RPB**	**Post-RPB**	**Pre-RPB**	**Post-RPB**
	1.4 (3/216)	0.7 (1/152)	8.3 (18/216)	4.6 (7/152)
	Cases excluding those not performed (*n* = 364)		Cases excluding those not performed (*n* = 343)	
	Failed in 3.6% (13/364)		Failed in 14.9% (51/343)	
	**Pre-RPB**	**Post-RPB**	**Pre-RPB**	**Post-RPB**
**Prior to plaque**	3.6 (1/28)	-	12.0 (3/25)	-
**Same day plaque**	4.9 (9/185)	-	15.0 (26/173)	-
**Within 1 month**	-	2.8 (2/71)	-	13.4 (9/67)
**2–3 months**	-	1.4 (1/72)	-	17.1 (12/70)
**3–6 months**	-	0/5	-	0/5
**More than 6 months**	-	0/3	-	1/3
***p*** **-value**	0.252 ^ⱡ^		0.372 *	

Statistical comparisons were made between Pre-RPB and Post-RPB: ^ⱡ^ Fisher’s Exact test, * Chi-squared test.

**Table 3:** Histology and genetic results of study patients stratified with respect to pre- and post-ruthenium plaque brachytherapy.

	Overall n (% Total)	Pre-RPB n (% Group)	Post-RPB n (% Group)	*p*-Value
**Cytomorphology**				
**Epithelioid or mixed**	101/364 (27.7)	57/213 (26.8)	44/151 (29.1)	0.520 *
Spindle	247/364 (67.9)	145/213 (68.1)	102/151 (67.5)	
**Melanoma of unspecified cell type**	3/364 (0.8)	1/213 (0.5)	2/151 (1.3)	
N/A	13/364 (3.6)	10/213 (4.7)	3/151 (2.0)	
**Genetic test undertaken**				
MLPA	52/368 (14.1)	26/216 (12.0)	26/152 (17.1)	0.177 *
MSA	291/368 (79.1)	172/216 (79.6)	119/152 (78.3)	
**Failed due to insufficient material**	25/368 (6.8)	18/216 (8.3)	7/152 (4.6)	
**Chromosome 1p**				
Loss	8/51 (15.7)	7/26 (26.9)	1/25 (4.0)	0.073 *
Disomy	35/51 (68.6)	16/26 (61.5)	19/25 (76.0)	
N/A	8/51 (15.7)	3/26 (11.5)	5/25 (20.0)	
**Chromosome 3**				
Monosomy	74/343 (21.6)	58/198 (29.3)	16/145 (11.0)	<0.001 *
Disomy	210/343 (61.2)	108/198 (54.5)	102/145 (70.3)	
N/A	59/343 (17.2)	32/198 (16.2)	27/145 (18.6)	
**Chromosome 6p**				
Disomy	25/51 (49.0)	13/26 (50.0)	12/25 (48.0)	0.642 *
Gain	20/51 (39.2)	9/26 (34.6)	11/25 (44.0)	
N/A	6/51 (11.8)	4/26 (15.4)	2/25 (8.0)	
**Chromosome 6q**				
Loss	4/51 (7.8)	3/26 (11.5)	1/25 (4.0)	0.717 *
Disomy	35/51 (68.6)	18/26 (69.2)	17/25 (68.0)	
Gain	5/51 (9.8)	2/26 (7.7)	3/25 (12.0)	
N/A	7/51 (13.7)	3/26 (11.5)	4/25 (16.0)	
**Chromosome 8p**				
Loss	7/51 (13.7)	3/26 (11.5)	3/25 (12.0)	0.622 *
Disomy	30/51 (58.8)	15/26 (57.7)	15/25 (60.0)	
Gain	8/51 (15.7)	3/26 (11.5)	5/25 (20.0)	
N/A	7/51 (13.7)	5/26 (19.2)	2/25 (8.0)	
**Chromosome 8q**				
Disomy	26/51 (51.0)	15/26 (57.7)	11/25 (44.0)	0.440 *
Gain	21/51 (41.2)	10/26 (38.5)	11/25 (44.0)	
N/A	4/51 (7.8)	1/26 (3.8)	3/25 (12.0)	

Statistical comparisons were made between Pre-RPB and Post-RPB: * Chi-squared test. N/A = Not available.

## Data Availability

The data presented in this study are available on request from the corresponding author.

## Abbreviations

The following abbreviations are used in this manuscript:

CGH	Comparative genomic hybridization
CM	Choroidal melanomas
DNA	Deoxyribonucleic acid
FISH	Fluorescence in situ hybridization
FNB	Fine-needle aspiration biopsy
GEP	Gene expression profiling
LBD	Largest basal diameter
LOOC	Liverpool Ocular Oncology Centre
LUMPO3	Liverpool Uveal Melanoma Prognosticator Online version 3
MLPA	Multiplex ligation dependent probe amplification
MSA	Microsatellite analysis
NHS	National Health Service
PBR	Proton beam radiotherapy
RPB	Ruthenium-106 plaque brachytherapy
SRF	Subretinal fluid

## References

[1] Wu M., Yavuzyigitoglu S., Brosens E., Ramdas W.D., Kilic E., Rotterdam Ocular Melanoma Study Group (2023). Worldwide Incidence of Ocular Melanoma and Correlation with Pigmentation-Related Risk Factors. Investig. Ophthalmol. Vis. Sci..

[2] Damato B. (2018). Ocular treatment of choroidal melanoma in relation to the prevention of metastatic death—A personal view. Prog. Retin. Eye Res..

[3] Rietschel P., Panageas K.S., Hanlon C., Patel A., Abramson D.H., Chapman P.B. (2005). Variates of survival in metastatic uveal melanoma. J. Clin. Oncol..

[4] Krantz B.A., Dave N., Komatsubara K.M., Marr B.P., Carvajal R.D. (2017). Uveal melanoma: Epidemiology, etiology, and treatment of primary disease. Clin. Ophthalmol..

[5] Prescher G., Bornfeld N., Hirche H., Horsthemke B., Jockel K.H., Becher R. (1996). Prognostic implications of monosomy 3 in uveal melanoma. Lancet.

[6] Damato B., Duke C., Coupland S.E., Hiscott P., Smith P.A., Campbell I., Douglas A., Howard P. (2007). Cytogenetics of uveal melanoma: A 7-year clinical experience. Ophthalmology.

[7] Damato B., Dopierala J.A., Coupland S.E. (2010). Genotypic profiling of 452 choroidal melanomas with multiplex ligation-dependent probe amplification. Clin. Cancer Res..

[8] Harbour J.W. (2014). A prognostic test to predict the risk of metastasis in uveal melanoma based on a 15-gene expression profile. Methods Mol. Biol..

[9] Caines R., Eleuteri A., Kalirai H., Fisher A.C., Heimann H., Damato B.E., Coupland S.E., Taktak A.F. (2015). Cluster analysis of multiplex ligation-dependent probe amplification data in choroidal melanoma. Mol. Vis..

[10] Damato B., Eleuteri A., Taktak A.F., Coupland S.E. (2011). Estimating prognosis for survival after treatment of choroidal melanoma. Prog. Retin. Eye Res..

[11] Hope-Stone L., Brown S.L., Heimann H., Damato B., Salmon P. (2015). How do patients with uveal melanoma experience and manage uncertainty? A qualitative study. Psychooncology.

[12] Grixti A., Angi M., Damato B.E., Jmor F., Konstantinidis L., Groenewald C., Heimann H. (2014). Vitreoretinal Surgery for Complications of Choroidal Tumor Biopsy. Ophthalmology.

[13] Angi M., Kalirai H., Taktak A., Hussain R., Groenewald C., Damato B.E., Heimann H., Coupland S.E. (2017). Prognostic biopsy of choroidal melanoma: An optimised surgical and laboratory approach. Br. J. Ophthalmol..

[14] Sen J., Groenewald C., Hiscott P.S., Smith P.A., Damato B.E. (2006). Transretinal choroidal tumor biopsy with a 25-gauge vitrector. Ophthalmology.

[15] Hussain R.N., Damato B., Heimann H. (2023). Choroidal biopsies; a review and optimised approach. Eye.

[16] Banou L., Tsani Z., Arvanitogiannis K., Pavlaki M., Dastiridou A., Androudi S. (2023). Radiotherapy in Uveal Melanoma: A Review of Ocular Complications. Curr. Oncol..

[17] Augsburger J.J., Shields J.A., Folberg R., Lang W., O’Hara B.J., Claricci J.D. (1985). Fine needle aspiration biopsy in the diagnosis of intraocular cancer. Cytologic-histologic correlations. Ophthalmology.

[18] Singh A.D., Medina C.A., Singh N., Aronow M.E., Biscotti C.V., Triozzi P.L. (2016). Fine-needle aspiration biopsy of uveal melanoma: Outcomes and complications. Br. J. Ophthalmol..

[19] Chiam P.J., Coupland S.E., Kalirai H., Groenewald C., Heimann H., Damato B.E. (2014). Does choroidal melanoma regression correlate with chromosome 3 loss after ruthenium brachytherapy?. Br. J. Ophthalmol..

[20] Thornton S., Coupland S.E., Heimann H., Hussain R., Groenewald C., Kacperek A., Damato B., Taktak A., Eleuteri A., Kalirai H. (2020). Effects of plaque brachytherapy and proton beam radiotherapy on prognostic testing: A comparison of uveal melanoma genotyped by microsatellite analysis. Br. J. Ophthalmol..

[21] Cunha Rola A., Taktak A., Eleuteri A., Kalirai H., Heimann H., Hussain R., Bonnett L.J., Hill C.J., Traynor M., Jager M.J. (2020). Multicenter External Validation of the Liverpool Uveal Melanoma Prognosticator Online: An OOG Collaborative Study. Cancers.

[22] Chao A.N., Rose K., Racher H., Altomare F., Krema H. (2023). Cytogenetic Abnormalities for Predicting the Risk of Metastases in Choroidal and Ciliary Body Melanoma. Investig. Ophthalmol. Vis. Sci..

[23] Coupland S.E., Kalirai H., Ho V., Thornton S., Damato B.E., Heimann H. (2015). Concordant chromosome 3 results in paired choroidal melanoma biopsies and subsequent tumour resection specimens. Br. J. Ophthalmol..

[24] Wackernagel W., Tarmann L., Mayer C., Langmann G., Wedrich A. (2013). Genetic analysis of uveal melanoma by array comparative genomic hybridization before and after radiotherapy. Spektrum Augenheilkd..

[25] Hussain R.N., Kalirai H., Groenewald C., Kacperek A., Errington R.D., Coupland S.E., Heimann H., Damato B. (2016). Prognostic Biopsy of Choroidal Melanoma after Proton Beam Radiation Therapy. Ophthalmology.

[26] Dogrusoz M., Kroes W.G., van Duinen S.G., Creutzberg C.L., Versluis M., Bleeker J.C., Marinkovic M., Luyten G.P., Jager M.J. (2015). Radiation Treatment Affects Chromosome Testing in Uveal Melanoma. Investig. Ophthalmol. Vis. Sci..

[27] Gold A.S., Murray T.G., Markoe A.M., Ehlies F., Latiff A., Wildner A., Bermudez E. (2014). Uveal melanoma gene expression status post radiotherapy. Optom. Vis. Sci..

[28] Kiilgaard J.F., Tebering J.F. (2011). The Safety and Applicability of 25G Trans-vitreal Retino-choroidal Biopsies in Prognostic Testing of Malignant Choroidal Melanoma. Investig. Ophthalmol. Vis. Sci..

[29] Bagger M., Smidt-Nielsen I., Andersen M.K., Jensen P.K., Heegaard S., Andersen K.K., Friis S., Kiilgaard J.F. (2018). Long-Term Metastatic Risk after Biopsy of Posterior Uveal Melanoma. Ophthalmology.

[30] Bagger M.M. (2018). Intraocular biopsy of uveal melanoma Risk assessment and identification of genetic prognostic markers. Acta Ophthalmol..

